# Development and Validation of an Autophagy-Related LncRNA Prognostic Signature in Head and Neck Squamous Cell Carcinoma

**DOI:** 10.3389/fonc.2021.743611

**Published:** 2021-10-01

**Authors:** Lin Shen, Na Li, Qin Zhou, Zhanzhan Li, Liangfang Shen

**Affiliations:** Department of Oncology, Xiangya Hospital, Central South University, Changsha, China

**Keywords:** autophagy, head and neck squamous carcinoma, long non-coding RNA, overall survival, prognostic signature

## Abstract

Head and neck squamous cell carcinoma (HNSCC) is one of the greatest public challenges because of delayed diagnosis and poor prognosis. In this study, we established an autophagy-associated long non-coding (Lnc)RNA prognostic signature to assess the prognosis of HNSCC patients. The LncRNA expression profiles and clinical information of 499 HNSCC samples were available in The Cancer Genome Atlas. Autophagic LncRNAs were analyzed using Pearson correlation. A co-expression network showed the interactions between autophagic genes and LncRNAs. An autophagic LncRNAs prognostic signature, consisting of MYOSLID, AL139287.1, AC068580.1, AL022328.2, AC104083.1, AL160006.1, AC116914.2, LINC00958, and AL450992.2, was developed through uni- and multivariate Cox regressions. High- and low-risk groups were classified based on the median risk scores. The high-risk group had significantly worse overall survival according to Kaplan–Meier curve analysis. Multivariate Cox regression demonstrated that risk scores were a significant independent prognostic factor (hazard ratio = 1.739, 95% confidence interval: 1.460–2.072), with an area under the curve of 0.735. Principal component analysis distinguished two categories based on the nine-LncRNA prognostic signature. In conclusion, this novel autophagic LncRNA signature is an independent prognostic factor and may suggest novel therapeutic targets for HNSCC.

## Introduction

Head and neck squamous cell carcinomas (HNSCCs) are common tumors that rank eighth worldwide in terms of incidence and mortality. HNSCCs are epithelial carcinomas derived from the oral cavity, nasal cavity, larynx, hypopharynx, and pharynx (1). They have a heterogeneous etiology based on multistage progression, genetic alterations, and environmental factors (2). Excessive smoking, alcohol consumption, and human papillomavirus infections are known risk factors for HNSCC development (3, 4). The initial symptoms, such as nasal congestion, oral ulcers, sore throat, and hoarseness, mimic common illnesses and often lead to late diagnoses. Although the diagnostic and treatment modalities for HNSCCs are rapidly improving, the 5-year survival rate has not increased significantly in the past few years, and the prognosis remains poor (5).

Long non-coding RNAs (LncRNAs) are non-protein-coding transcribed RNAs with more than 200 base pairs (6). LncRNAs were previously regarded as “dark matter” and “transcriptional noise” without biofunctions, but recent studies have demonstrated that many LncRNAs are involved in important bioactivities, such as chromatin modification, transcriptional activation and interference, and cell differentiation and proliferation (7–9). LncRNAs are mostly found in the nucleus, particularly in the chromatin fraction, which underlines their regulatory role in gene transcription. Moreover, genome-wide tumor association studies have revealed that thousands of LncRNAs are associated with tumorigenesis and metastasis (10, 11). LncRNAs are considered novel biomarkers for guiding treatment due to recent advances in our understanding the molecular mechanisms underlying cancer-related LncRNAs (12, 13).

Autophagy is a physiological process that membrane-encloses damaged or degenerated proteins and organelles, and delivers them to lysosomes for degradation (14). Autophagic dysregulation is related to various diseases, including neurodegenerative, inflammatory, cardiovascular, and neoplastic disorders (15–18). Predictive functions of autophagy in various cancers are gradually being explored. An autophagy-related gene signature was recently reported to be closely related with HNSCC outcomes (19, 20). Another autophagic LncRNA signature was found to accurately predict the prognoses of bladder urothelial carcinomas (21). Although autophagic genes and LncRNAs can reportedly serve as HNSCC biomarkers, their prognostic value remains unclear. Our study aimed to clarify the prognostic functions of autophagy-associated LncRNAs in HNSCC.

## Methods

### Data Acquisition

Messenger RNA (mRNA) sequences and clinical data of HNSCC patients and controls (peritumor tissues) were acquired from The Cancer Genome Atlas (TCGA; https://cancergenome.nih.gov/). The inclusion criteria were HNSCC patients; complete LncRNA expression data and clinical information; and follow-up duration longer than 30 days. Complete clinical information, including age, sex, tumor grade, American Joint Committee on Cancer (AJCC) stage, TNM stage, and survival data, were downloaded for analysis. Autophagic genes were acquired from the Human Autophagy Database (http://autophagy.lu/index.html). Simple nucleotide variations of HNSCC were also downloaded from TCGA.

As our data were publicly available, no specific ethical approval or informed consent was required.

### Identification of Autophagic LncRNAs

LncRNA expression profiles of HNSCC patients were obtained from TCGA. All data were standardized using the limma package for R software (v.3.6.3; R Foundation for Statistical Computing, Vienna, Austria) before further analysis. Pearson correlation analyses were performed on LncRNAs and autophagic genes in HNSCC patients using R software (v.3.6.3). A correlation coefficient (*R*) > 0.3 and *p*-value < 0.001 were considered significant for autophagic LncRNAs. A co-expression network between autophagic LncRNAs and genes was also built using Cytoscape (v.3.8.2).

### Establishment of Prognostic Signature

Uni- and multivariate Cox regression analyses were performed to establish potential prognostic signatures. First, the association between autophagic LncRNAs and survival rates was assessed by univariate Cox regression. *p* < 0.01 was regarded significant for prognosis-related LncRNAs in HNSCC patients. Multivariate Cox regression analysis was then performed for the selected prognostic LncRNAs. A risk-score-based prognostic signature was computed as follows: risk score = lncRNA1_β×Expression_ + lncRNA2_β×Expression_ + lncRNA(N)_β×Expression_ (22).

### Prognosis Prediction

According to the formula above, HNSCC patients were classified into high- and low-risk groups based on median risk scores. A Kaplan–Meier curve was plotted to compare survival between groups using the two-sided log-rank test. Uni- and multivariate Cox regressions were performed to evaluate the effect of clinical variables on survival in HNSCC patients and to determine if the risk scores were independent prognostic factors. Predictive accuracy was determined by calculating the area under the receiver operating characteristic (ROC) curve (AUC). We also investigated the association of the expression level of each autophagic LncRNA with overall survival (OS) in HNSCC patients using Kaplan–Meier curves. To clarify the impact of single autophagy-related LncRNAs on HNSCC prognosis, we assessed their associations with the various clinical characteristics using Student’s *t*-test or one-way analysis of variance (ANOVA).

### Functional Analysis

Principal component analysis (PCA) was performed to determine similarities and differences between the autophagic-LncRNA and whole expression profiles of HNSCC patients. Functional enrichment was assessed using gene set enrichment analysis (GSEA; v.4.0.3; http://www.broadinstitute.org/gsea/index.jsp). We verified whether or not differentially expressed genes between high- and low-risk groups were enriched in autophagy-related processes.

### Prognostic Signature Validation

We detected the expression of nine autophagic LncRNAs in 190 HNSCC patients, which were used for prognostic signature validation. The validation data were provided by the Ethics Committee of Xiangya Hospital, Central South University. All HNSCC cases were pathologically confirmed; the clinical characteristics are shown in **Table S1**.

The expression levels of target LncRNAs were measured using real-time polymerase chain reaction (RT-PCR). Total RNA was extracted from the tissue specimens using the GeneJET RNA purification kit (Thermo Fisher Scientific, Waltham, MA, USA) according to manufacturer’s instructions. Complementary DNAs (cDNAs) were synthesized using SuperScript III Reverse Transcriptase (Invitrogen; Thermo Fisher Scientific). LncRNA expression was assessed by RT-PCR ([model]; Bio-Rad Laboratories Inc., Hercules, CA, USA). Expression levels were quantified using the 2^−ΔΔCt^ method.

### Statistical Analysis

All statistical analyses were performed using R software (v.4.0.5). Survival probabilities were compared between groups using Kaplan–Meier curve analysis. The diagnostic accuracy of the signature was evaluated by ROC curve analysis. Nomographs were plotted to estimate the 1-, 3-, and 5-year OS rates of individuals according to different risk scores and clinical parameters. Pearson and Spearman correlation analyses were performed. Simple nucleotide variations were analyzed using the maftools R package. *p* < 0.05 was considered significant.

## Results

### Identification of Prognostic Autophagy-Related LncRNAs

**Figure 1:**
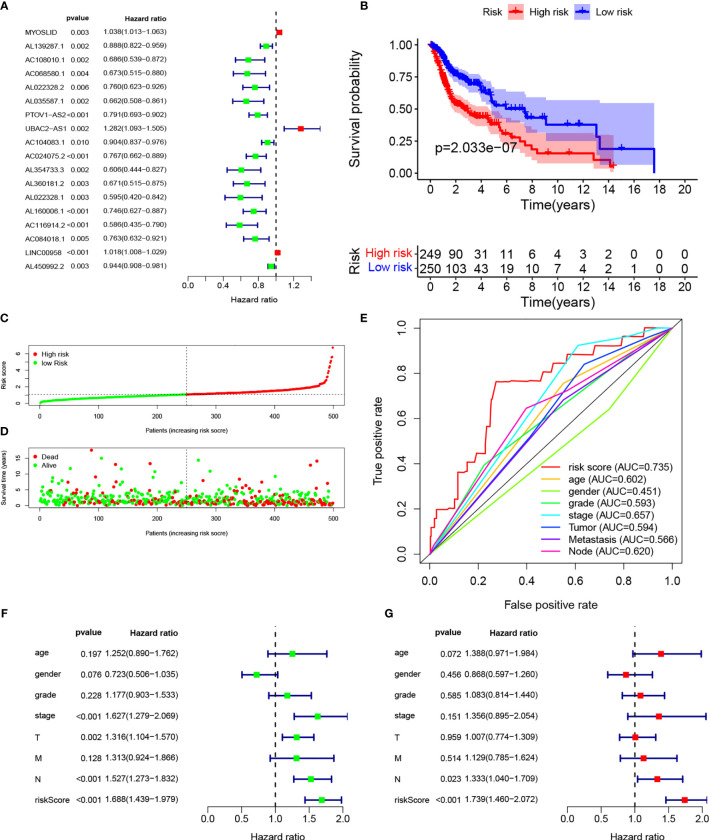
Establishment of autophagy-related lncRNA signature for HNSCC patients. **(A)** Forest plot of univariate Cox regression for autophagic lncRNAs correlated with HNSCC prognosis. **(B)** Kaplan–Meier curves of overall survival for high- and low-risk groups. **(C)** Risk scores of the high- and low-risk groups. **(D)** Scatterplot of risk scores and survival time/survival outcomes. **(E)** ROC curves of prognostic signature and other clinical parameters. **(F, G)** Forest plots of univariate and multivariate Cox regressions, respectively, for association between risk score and overall survival.

We identified 14,142 LncRNAs by RNA-sequence analysis of HNSCC patients from TCGA. We also obtained 257 autophagy-related genes from a public database (**Table S2**). In total, 910 autophagy-related LncRNAs met the criteria (*R* > 0.3 and *p* < 0.001). Cox regression analyses were then performed to determine the autophagy-related LncRNAs with potential prognostic value for HNSCC (**Table S3**). Of the 910 LncRNAs, 18 were linked with the OS of HNSCC patients. Multivariate Cox regression showed that 9 of those 18 LncRNAs (MYOSLID, AL139287.1, AC068580.1, AL022328.2, AC104083.1, AL160006.1, AC116914.2, LINC00958, and AL450992.2) were involved in the prognostic signature (**Figures 1A**, **S1A–I** and **Table S4**).

### Establishment of the Nine-LncRNA Prognostic Signature

The risk score of the HNSCC patients was calculated as follows: risk score = (0.0236 × Exp_MYOSLID_) + (−0.0890 × Exp_AL139287.1_) + (−0.3069 × Exp_AC068580.1_) + (0.2869 × Exp_AL022328.2_) + (−0.0802 × Exp_AC104083.1_) + (−0.2112 × Exp_AL160006.1_) + (−0.4007 × Exp_AC116914.2_) + (0.0140 × Exp_LINC00958_) + (−0.0425 × Exp_AL450992.2_). The prognostic value of this nine-LncRNA risk signature for HNSCC patients was evaluated. Based on the median risk scores, 249 and 205 HNSCC patients were classified as high and low risk, respectively. Kaplan–Meier curve analysis revealed significant differences in OS between the groups; OS was worse in the high-risk group (**Figure 1B**). We ranked the HNSCC patients according to their risk scores based on the nine-LncRNA prognostic signature (**Figure 1C**). The scatter diagram demonstrated that the survival rates of the HNSCC patients were correlated with the risk scores; the mortality rate increased with an increased risk score (**Figure 1D**). The AUC value of the nine lncRNAs was 0.735. The AUC values for age, sex, tumor grade, AJCC stage, T stage, N stage, and M stage were 0.602, 0.451, 0.593, 0.657, 0.594, 0.620, and 0.566, respectively (**Figure 1E**). These results confirmed that the nine-LncRNA prognostic signature could predict the survival outcomes of HNSCC patients.

### The LncRNA Signature Was Independently Associated With Prognosis

Multivariate Cox regression based on risk scores and clinical characteristics was performed to determine whether the nine-LncRNA prognostic signature was an independent prognostic factor. Univariate Cox regression demonstrated that the risk score was significantly correlated with OS (**Figure 1F**). Multivariate Cox regression also showed a significant association between the risk score and OS in HNSCC patients (**Figure 1G**).

### Clinical Significance of the Prognostic Signature

**Figure 2:**
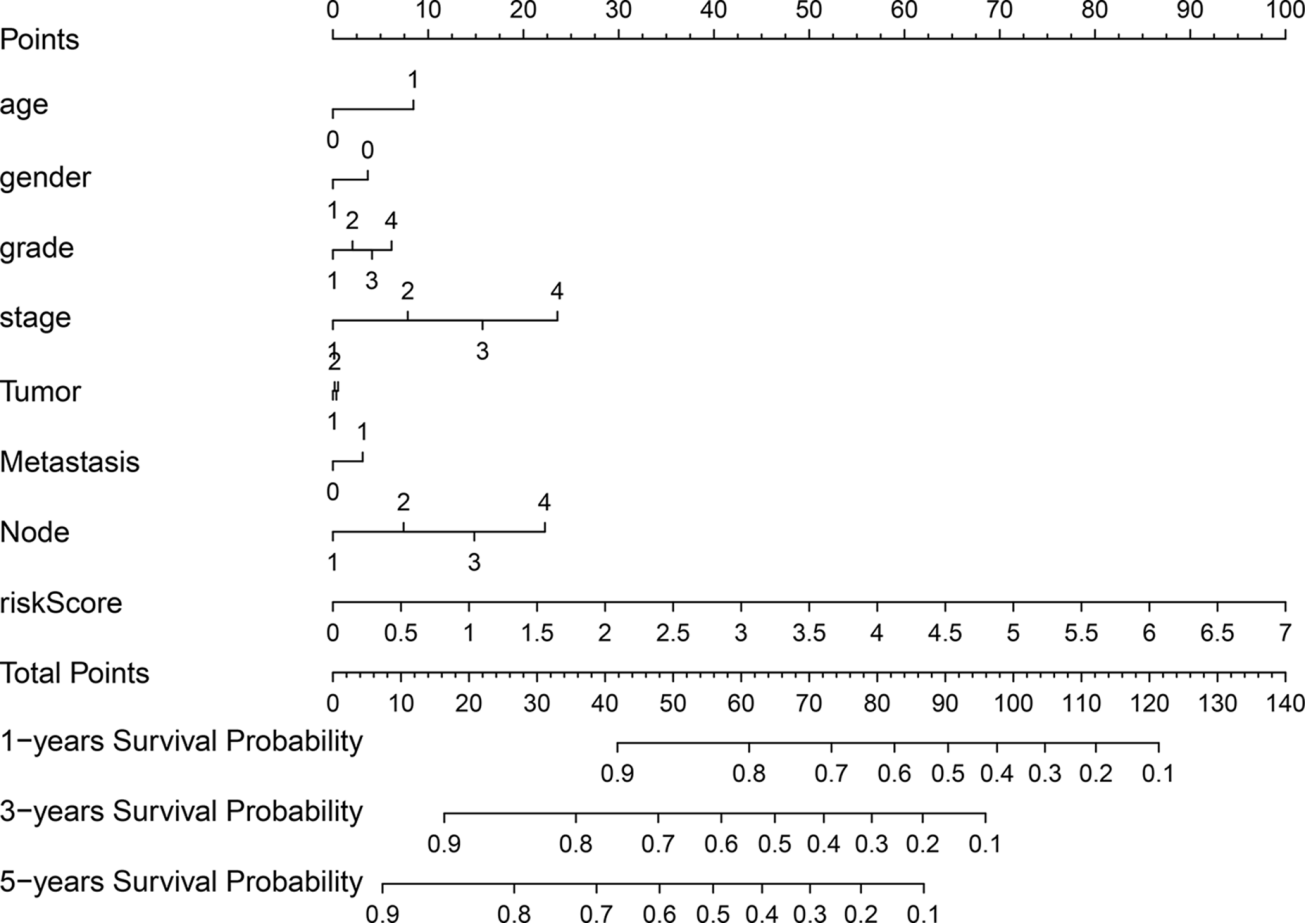
Nomograph of 1-, 3-, and 5-year overall survival probabilities predicted based on autophagy-related LncRNA signature.

**Figure 3:**
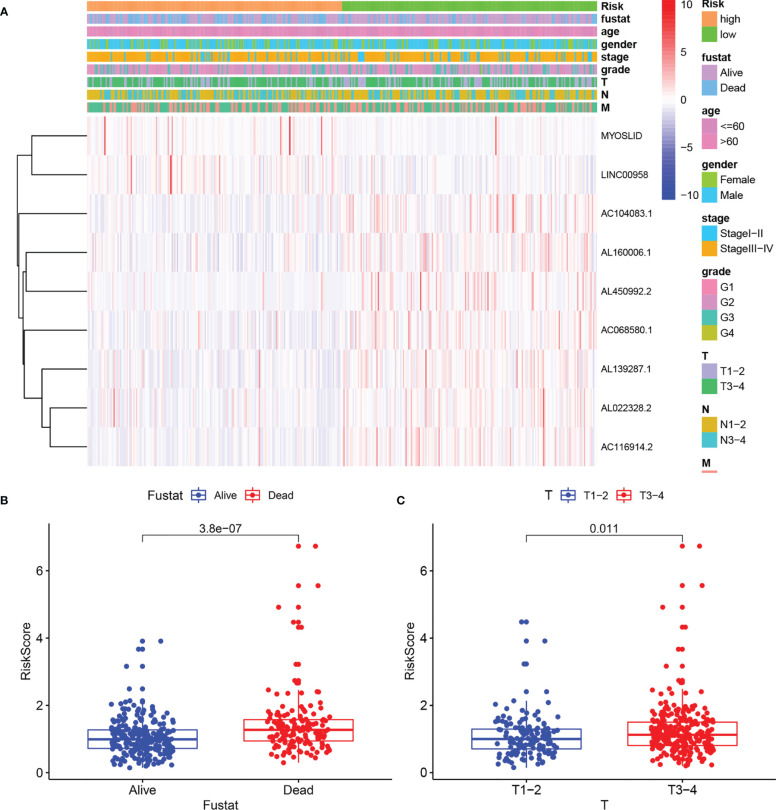
Correlation of risk score with clinical parameters. **(A)** Heatmaps of clinical parameters and autophagy-related LncRNAs between high- and low-risk groups. **(B)** Boxplot of risk score difference between alive and dead groups. **(C)** Boxplot of risk score difference between T stage1–2 and T stage 3–4.

**Figure 4:**
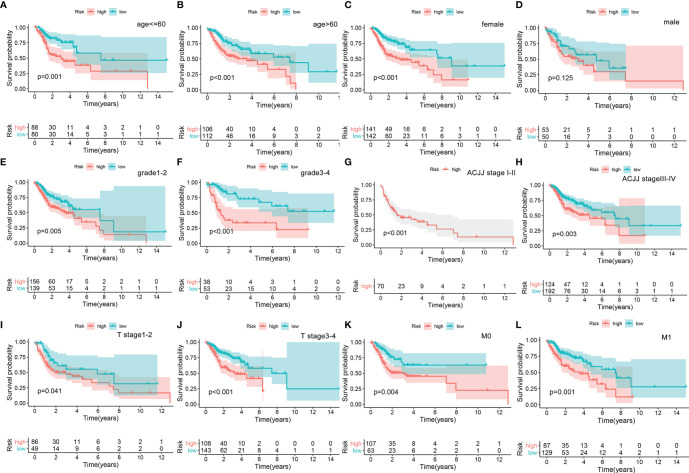
Overall survival difference between high- and low-risk groups for HNSCC patients stratified by clinical parameters, including age [age ≤60, ag e>60, **(A, B)**], gender [female, male, **(C, D)**], grade [G1–2, G3–4, **(E, F)**], AJCC stage [Stage I–II, Stage III–IV, **(G, H)**], T stage [Stage 1–2, Stage 3–4, **(I, J)**], and M stage [M0, M1, **(K, L)**].

A nomogram was plotted to evaluate 1-, 3-, and 5-year survival based on the risk score of the prognostic signature and clinical data. The nomogram demonstrated that the risk score was the most significant contributor to the 3- and 5-year OS of HNSCC patients (**Figure 2**). We then investigated the associations of high- and low-risk status with clinical parameters using the Chi-square test. No significant differences in clinical parameters were observed between groups (*p* > 0.05; **Figure 3A**). We also analyzed risk scores according to clinical parameters and found significant differences in relation to survival status and T stage. Patients with poor survival status and advanced T stage had higher risk scores (*p* < 0.05; **Figures 3B, C**). To verify the utility of the prognosis signature, we also performed subgroup analyses based on age (≤ 60 vs. > 60 years; **Figures 4A, B**), sex (**Figures 4C, D**), grade (T1–2 vs. T3–4; **Figures 4E, F**), AJCC stage (stage I–II vs. stage III–IV; **Figures 4G, H**), T stage (T1–2 vs. T3–4; **Figures 4I, J**), and M stage (M0 vs. M1; **Figures 4K, L**). We found that the prognostic signature was related to OS in all strata of the population. The high-risk group had worse OS than the low-risk group.

In addition, we investigated LncRNA expression levels according to the various clinical characteristics. The expression levels of AC116914.2 and AL022328.2 were higher in males compared to females (**Figure S2A**). The expression levels of AL022328.2 and AL450992.2 were higher for higher grades, but the expression of MYOSLID had no correlation with grade (**Figure S2B**). No significant differences were found in expression levels by AJCC stage (**Figure S2C**). The expression levels of AC068580.1, LINC00958, and MYOSLID increased from T1 to T3, but were decreased for T4 (**Figure S2D**). The results regarding the N stage were similar to those for the AJCC stages (**Figure S2E**). In terms of the M stage, AL022328.2 and AL139287.1 expression levels were higher, but AC116914.2 expression was lower, in the M1 stage (**Figure S2F**).

### Gene Mutations

**Figure 5:**
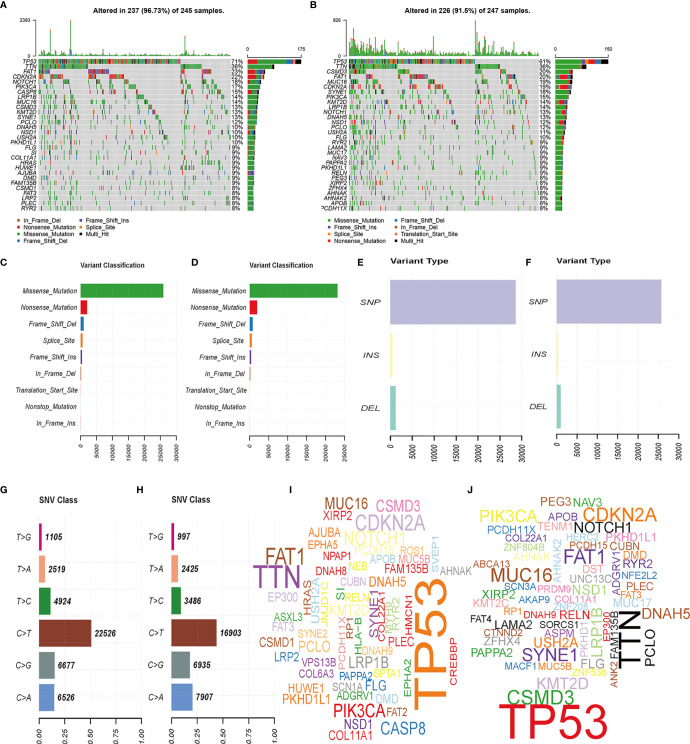
Landscape of mutation profiles between high- and low-risk HNSCC patients. **(A, B)** Waterfall plots of mutation information in each sample. **(C, D)** Variant classification. **(E, F)** Distribution of genetic alterations. **(G, H)** SNV classes. **(I, J)** Gene clouds of mutation frequencies in HNSCC patients.

We analyzed the gene mutation profiles of 492 HNSCC patients based on the risk scores. The high- and low-risk groups consisted of 245 (49.1%) and 247 (49.5%) samples, respectively, while 7 (1.4%) samples were excluded because of a lack of mutation data. Waterfall plots were used to evaluate the genes of the patients in the two groups (**Figures 5A, B**). The top 10 mutated genes in the high-risk group were TP53, TTN, FAT1, CDKN2A, NOTCH1, PIK3CA, CASP8, LRP1B, MUC16, and CSMD3. Although some mutated genes overlapped between groups, several genes were more frequent in the high-risk group, including TP53 (*p* = 0.004), HRAS (*p* = 0.001), and CASP8 (*p* = 0.001). Missense mutations accounted for most of the mutations in both groups (**Figures 5C, D**). The single-nucleotide variant was the most common type (**Figures 5E, F**), and C-to-T transversions were the most common single-nucleotide variant (**Figures 5G, H**). The gene cloud plots showed the top mutated genes in the two groups (**Figures 5I, J**).

### Co-Expression Network of Autophagy-Related LncRNAs and mRNAs

**Figure 6:**
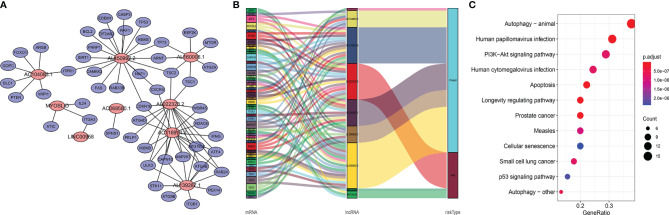
Functional annotations of autophagic LncRNAs prognostic signature as per co-expressed mRNA. **(A)** Co-expressed regulatory network of LncRNAs-mRNA based on the signature. **(B)** Sankey diagram of co-occurrences of LncRNAs, mRNAs, and factors. **(C)** KEGG enrichment analysis of co-expressed mRNAs related with the LncRNA signature.

**Figure 7:**
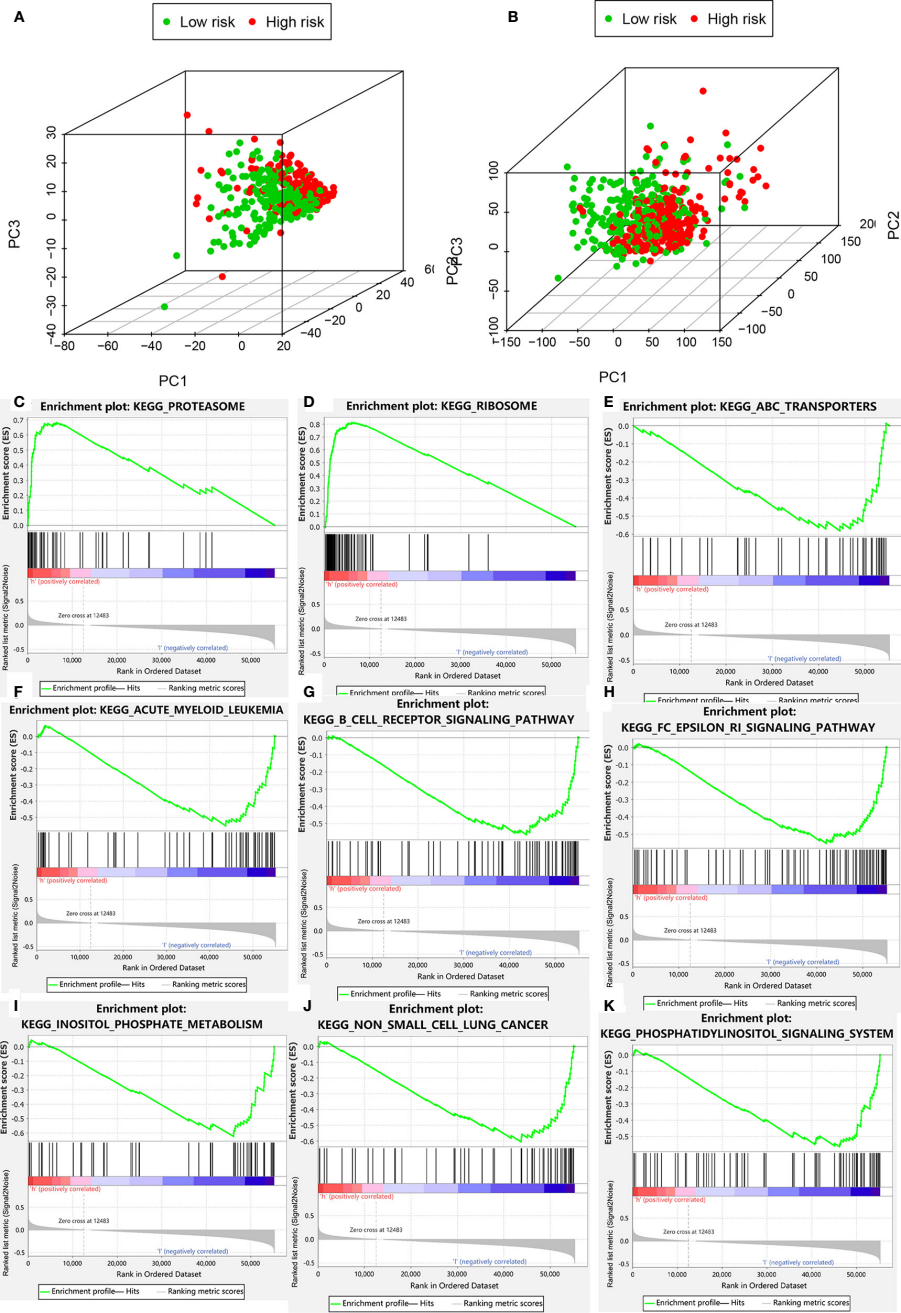
Clustering analysis based on risk score. **(A)** PCA of two categories. **(B)** PCA for genome-wide expression profiles between high- and low-risk groups. **(C–K)** Enrichment plot for KEGG pathways analysis.

Studies have suggested that mutual regulation between LncRNAs and mRNAs is critical for tumor progression. We established a co-expression network using Cytoscape. There were 48 mRNAs associated with nine target LncRNAs (*R* > 0.3, *p* < 0.001; **Figure 6A**). Associations among co-expressed mRNAs and LncRNAs in the prognostic signature and risk types were visualized using a Sankey diagram (**Figure 6B**). AL022328.2 was the major component of overall risk, while MYOSLID and LINC00958 accounted for small proportions (**Figure 7B**). The corresponding mRNAs were ATF4, ATG16L2, ATG4B, ATG4D, CAPN10, CDKN1B, HDAC6, IKBKB, ITGA3, MAP2K7, PELP1, RAB24, TSC1, TSC2, ULK3, and WDR45. Among these mRNAs, CAPN10, which is involved in degradation of the extracellular matrix and nitric oxide synthase signaling, was most strongly correlated with AL022328.2. We performed Kyoto Encyclopedia of Genes and Genomes (KEGG) enrichment analysis to identify the co-expressed mRNAs most associated with autophagic LncRNAs, and determined that the top five enriched signaling pathways were involved in autophagy, human papillomavirus infection, PI3K–Akt pathway, human cytomegalovirus infection, and apoptosis (**Figure 6C**).

### Functional Analysis

PCA was performed to determine differences in gene distribution between the high- and low-risk groups. No significant differences were found in the whole gene expression profiles of the two groups (**Table S5**), but significant differences were observed within the autophagic-LncRNA set (**Figures 7A, B**). GSEA was used to investigate the functional enrichment of genes. We analyzed 178 gene sets and found that 14 and 164 were upregulated in the high-risk (**Table S6**) and low-risk (**Table S7**) groups, respectively. KEGG pathway analysis revealed that the proteasome pathways and ribosomes were significantly enriched in the high-risk group (**Figures 7C, D**). ATP-binding cassette transporters (**Figure 7E**), acute myeloid leukemia (**Figure 7F**), B-cell receptor pathway (**Figure 7G**), FC epsilon RI pathway (**Figure 7H**), inositol phosphate metabolism (**Figure 7I**), non-small cell lung cancer (**Figure 7J**), and the phosphatidylinositol system (**Figure 7K**) were highly enriched in the low-risk group.

### LncRNA Expression Levels

**Figure 8:**
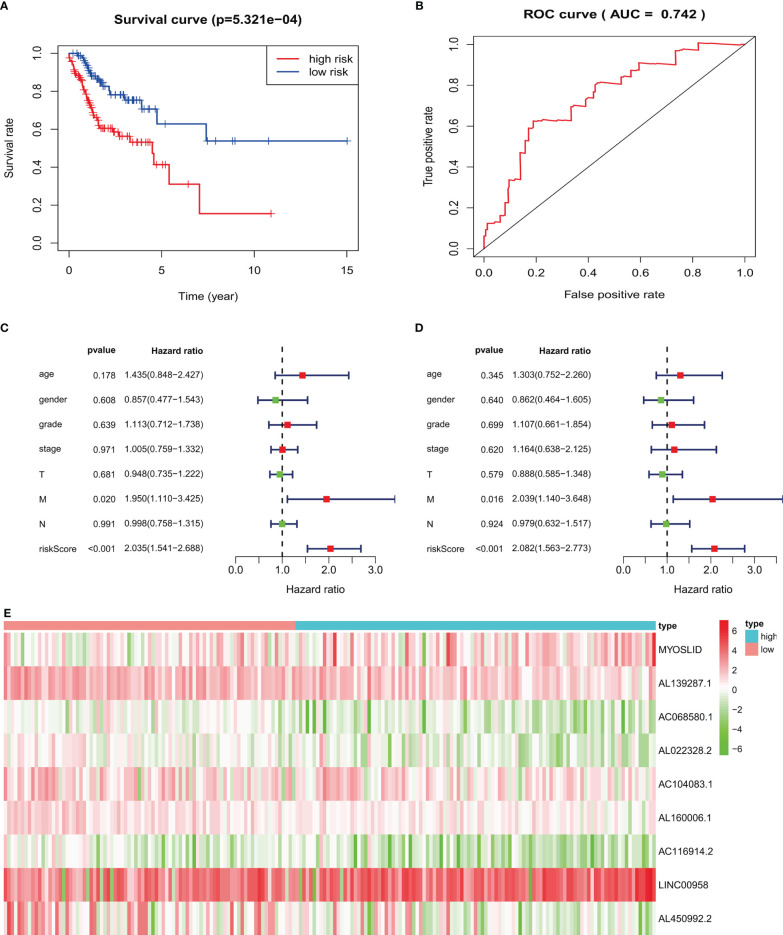
Validation of prognostic signature in an independent HNSCC population: **(A)** high-risk HNSCC patients have poorer overall survival. **(B)** ROC curves of prognostic signature in validated HNSCC patients. **(C, D)** Forest plots of univariate and multivariate Cox regression, respectively, about association between risk score and overall survival in validated HNSCC patients. **(E)** Heatmap of nine target LncRNAs in high-risk and low-risk groups.

The expression levels of nine LncRNAs were compared between 502 tumor tissue and 44 normal tissue specimens from TCGA (**Figure S3A**). The results showed that MYOSLID, LINC00958, and AL022328.2 were expressed more, while AL450992.2 and AC068580.1 were expressed less, in tumor compared to normal tissues (**Figure S3B**). These results were consistent with our analysis. However, AL104083.1 and AC116914.2 were significant risk factors in multivariate analysis. AL139287.1 and AL160006.1 showed no significant differences between tumor and normal tissues. The expression levels of the nine lncRNAs are presented in **Figure 8E**.

### Validation of Prognostic Signature

For HNSCC data validation, we analyzed 190 HNSCC patients, separated into high- and low-risk groups based on the risk score. The results indicated that the high-risk group had worse OS than the low-risk group (*p* < 0.05; **Figure 8A**). The AUC value of the validated data was 0.742 (**Figure 8B**). Univariate Cox regression demonstrated that risk scores (hazard ratio [HR] = 2.035, 95% confidence interval [CI]: 1.541–2.688, *p* < 0.001, **Figure 8C**) and M stage (HR = 1.950, 95% CI: 1.110–3.425, *p* = 0.020) were related to poor OS. Multivariate Cox regression demonstrated that risk scores (HR = 2.082, 95% CI: 1.563–2.773, *p* < 0.001, **Figure 8D**) and M stage (HR = 2.039, 95% CI: 1.140–3.648, *p* = 0.016) were independently correlated with OS.

## Discussion

Head and neck cancers are among the most common malignancies worldwide, and about 90% of these are squamous cell carcinomas (23). Surgery combined with chemoradiotherapy provides favorable outcomes in early-stage HNSCC (24, 25). However, early-stage HNSCC patients usually have no obvious symptoms. Most patients are diagnosed at moderate or advanced stages, and about 17% of patients miss the window for surgery. Advanced HNSCCs have a poor prognosis and high recurrence rates (26). Therefore, there is an urgent need to identify potential prognostic biomarkers. Many reports have suggested that biomarkers identified through database mining may predict HNSCC prognosis (27–29). Autophagy can remove harmful substances from the body and keep the internal environment stable (30). However, autophagy can also promote tumor growth by providing energy. LncRNAs have been widely investigated as autophagy-related regulators of tumorigenesis (31).

Autophagy is closely related with oncogenesis and is important in the treatment and prognosis of various cancers (32). In the oncogenesis stage of HNSCC, smoking can induce autophagy and lead to oxidative stress (33). Moreover, knockdown of essential autophagy genes and biochemical inhibition of autophagy can remarkably enhance HPV infectivity (34). During treatment, autophagy is known to be correlated with chemo- and radioresistance due to autophagy-mediated cell death or survival (35). Autophagy is also significantly related to HNSCC prognosis; for example, an autophagic gene signature is reportedly a strong predictor of HNSCC prognosis (27). LncRNAs are increasingly being considered as novel biomarkers and prognostic markers of cancers. Autophagy-related LncRNA signatures can also predict the prognosis of colon adenocarcinoma and breast cancer (36, 37). However, there are no reports on the predictive potential of autophagic LncRNA signatures for HNSCC. Therefore, this study was performed to evaluate the role of autophagic LncRNAs in HNSCC.

We identified a prognostic signature based on nine LncRNAs, namely, MYOSLID, AL139287.1, AC068580.1, AL022328.2, AC104083.1, AL160006.1, AC116914.2, LINC00958, and AL450992.2, to predict OS in HNSCC patients. Among them, AL139287.1, AC068580.1, AC104083.1, AL160006.1, AC116914.2, and AL450992.2 were protection-related, while MYOSLID, AL022328.2, and LINC00958 were risk-related, based on the Sankey diagram. MYOSLID reportedly promotes invasion and metastasis by regulating the partial epithelial–mesenchymal transition in HNSCCs (38). LINC00958 plays a role in multiple cancers by upregulating the microRNA-625/NUAK pathway and contributes to nasopharyngeal carcinomas (39). LINC00958 regulates the miR-627-5p/YBX2 axis to facilitate cell proliferation and migration in oral squamous cell carcinoma (40). In this study, a co-expression network between these nine LncRNAs and the autophagic genes with which they interact was used to determine the mechanisms potentially underlying the autophagy-related LncRNA signature and HNSCC prognosis. The risk score increased as the expression levels of the three risk-related LncRNAs increased and those of the six protection-related LncRNAs decreased. Kaplan–Meier curve analysis revealed that the high-risk group had significantly poorer OS. The AUC value was 0.735, which indicates the reliability and stability of the prognostic signature. In addition, analysis of single autophagy-related LncRNAs showed that higher expression levels of two risk-related LncRNAs were associated with a poor prognosis, while higher levels of the remaining LncRNAs were related to a better prognosis. Multivariate Cox regression demonstrated that the autophagic LncRNA signature is an independent prognostic factor (*p* < 0.001). PCA of whole gene expression profile data revealed no significant differences between groups, but significant differences were seen when analyzing the autophagy-related LncRNA set. GSEA demonstrated that the 14 autophagy-related gene sets, which mainly participate in proteasome and ribosome pathways, were more common in the high-risk group. Proteasomes constitute a degradation system for oxidatively damaged proteins and are involved in cancer development because the ubiquitin–proteasomal system is a key regulator of various molecular pathways (41). Ribosomes are required to convert the information contained in mRNAs into functional proteins; therefore, promoting ribosome and protein synthesis to maintain tumor cell growth and division is essential (42). More importantly, both of these pathways may be involved in autophagy (43, 44). Autophagy-related genes enriched in these pathways may shed light on the mechanisms underlying the poor prognosis of the high-risk group. Autophagy is associated with immune filtration in tumor patients (45). Considering the important role of immune functions, future studies should investigate immune changes in HNSCC patients.

There were several limitations to this study. First, HNSCC encompasses several types of cancers, each of which require separate, detailed analyses. The present risk model was based on a public database, so validation with larger samples is required. Finally, additional experiments are required to elucidate the molecular mechanisms and potential treatment targets.

Our analyses highlight the prognostic value of the nine-LncRNA signature for HNSCC patients, which could guide clinical decisions and treatment plans, and thus improve prognosis.

## Data Availability Statement

The datasets presented in this study can be found in online repositories. The names of the repository/repositories and accession number(s) can be found in the article/**Supplementary Material**.

## Ethics Statement

The studies involving human participants were reviewed and approved by the Ethics Committee of Xiangya Hospital, Central South University (201404355). The patients/participants provided their written informed consent to participate in this study.

## Author Contributions

ZL designed this study and directed the research group in all aspects, including planning, execution, and analysis of the study. LinS drafted the manuscript. NL and QZ collected the data. ZL provided the statistical software, performed the data analysis, and arranged the figures and tables. ZL and LiaS revised the manuscript. All authors contributed to the article and approved the submitted version.

## Funding

This study was supported by the National Natural Science Foundation of China (No. 82003239), the Hunan Province Natural Science Foundation (Youth Foundation Project) (No. 2019JJ50945), and the Science Foundation of Xiangya Hospital for Young Scholar (No. 2018Q012).

## Conflict of Interest

The authors declare that the research was conducted in the absence of any commercial or financial relationships that could be construed as a potential conflict of interest.

## Publisher’s Note

All claims expressed in this article are solely those of the authors and do not necessarily represent those of their affiliated organizations, or those of the publisher, the editors and the reviewers. Any product that may be evaluated in this article, or claim that may be made by its manufacturer, is not guaranteed or endorsed by the publisher.

## Supplementary Material

Supplementary Figure 1 Kaplan-Meier curves of associations between signature lncRNAs and prognosis in HNSCC patients. **(A)** MYOSLID; **(B)** LINC00958; **(C)** AL450992.2; **(D)** AL160006.1; **(E)** AL139287.1; **(F)** AL022328.2; **(G)** AC116914.2; **(H)** AC104083.1; **(I)** AC068580.1.

Supplementary Figure 2 Relation between autophagic signature lncRNAs and clinical parameters. **(A)** gender (female vs. male); **(B)** grade (G1, G2, G3, G4); **(C)** AJCC Stage (Stage I, II, III, IV); **(D)** T stage (T1, T2, T3, T4); **(E)** Stage N (N0, N1, N2, N3); **(F)** Stage M [M0, M1, MX (unknown)].

Supplementary Figure 3 Expression levels of signature lncRNAs between normal tissues (n=44) and tumor tissues (n=502). **(A)** heatmap and **(B)** boxplot.

Supplementary File S1 Original data.
