# Supplementary figures and images for: Development and Validation of an Autophagy-Related LncRNA Prognostic Signature in Head and Neck Squamous Cell Carcinoma
Source: Front Oncol. 2021 Oct 1;11:743611. doi: 10.3389/fonc.2021.743611 (PMC8517509; doi:10.3389/fonc.2021.743611)

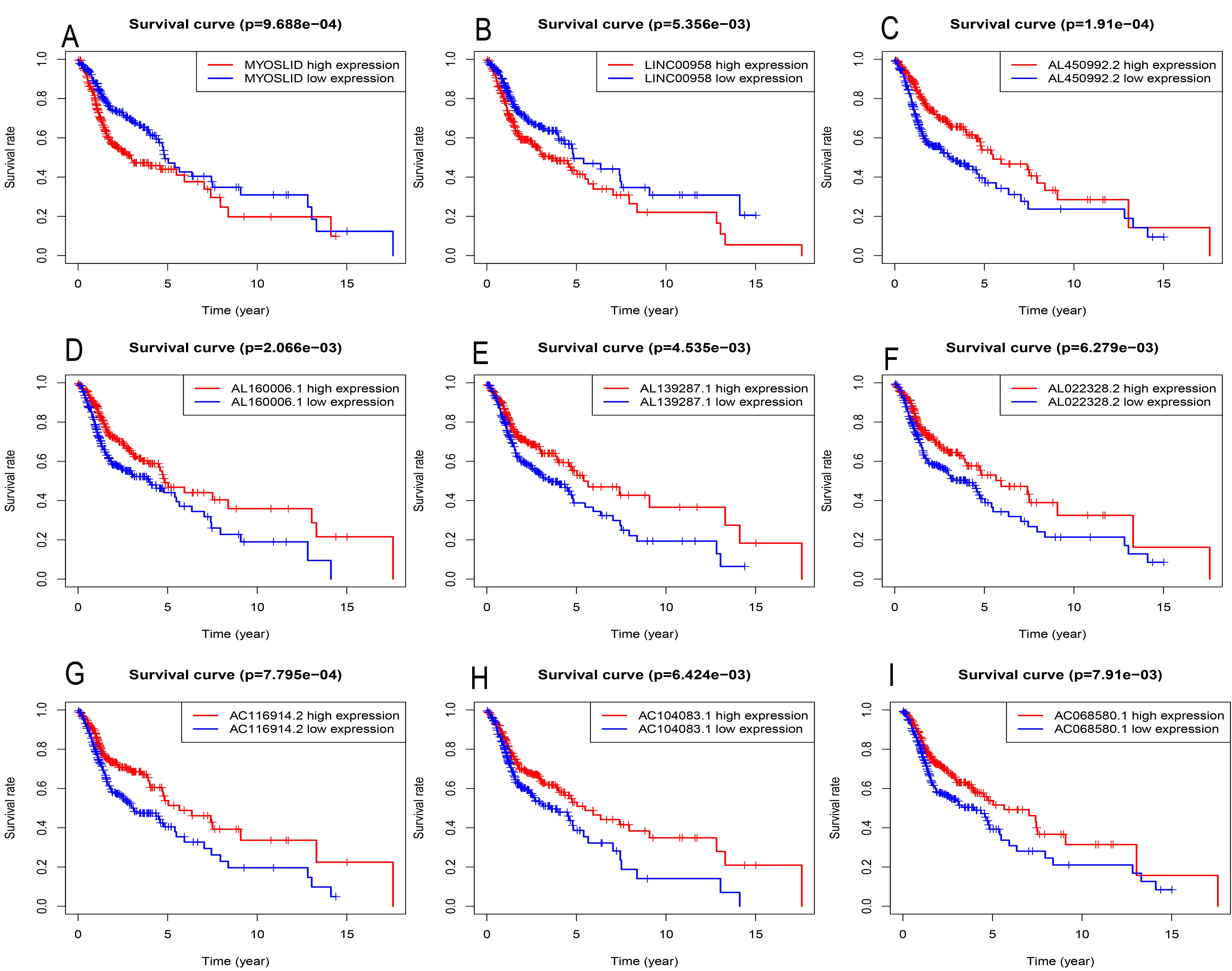

Supplement: Supplementary Figure 1 — Kaplan-Meier curves of associations between signature lncRNAs and prognosis in HNSCC patients. (A) MYOSLID; (B) LINC00958; (C) AL450992.2; (D) AL160006.1; (E) AL139287.1; (F) AL022328.2; (G) AC116914.2; (H) AC104083.1; (I) AC068580.1. [file Image_1.tif]

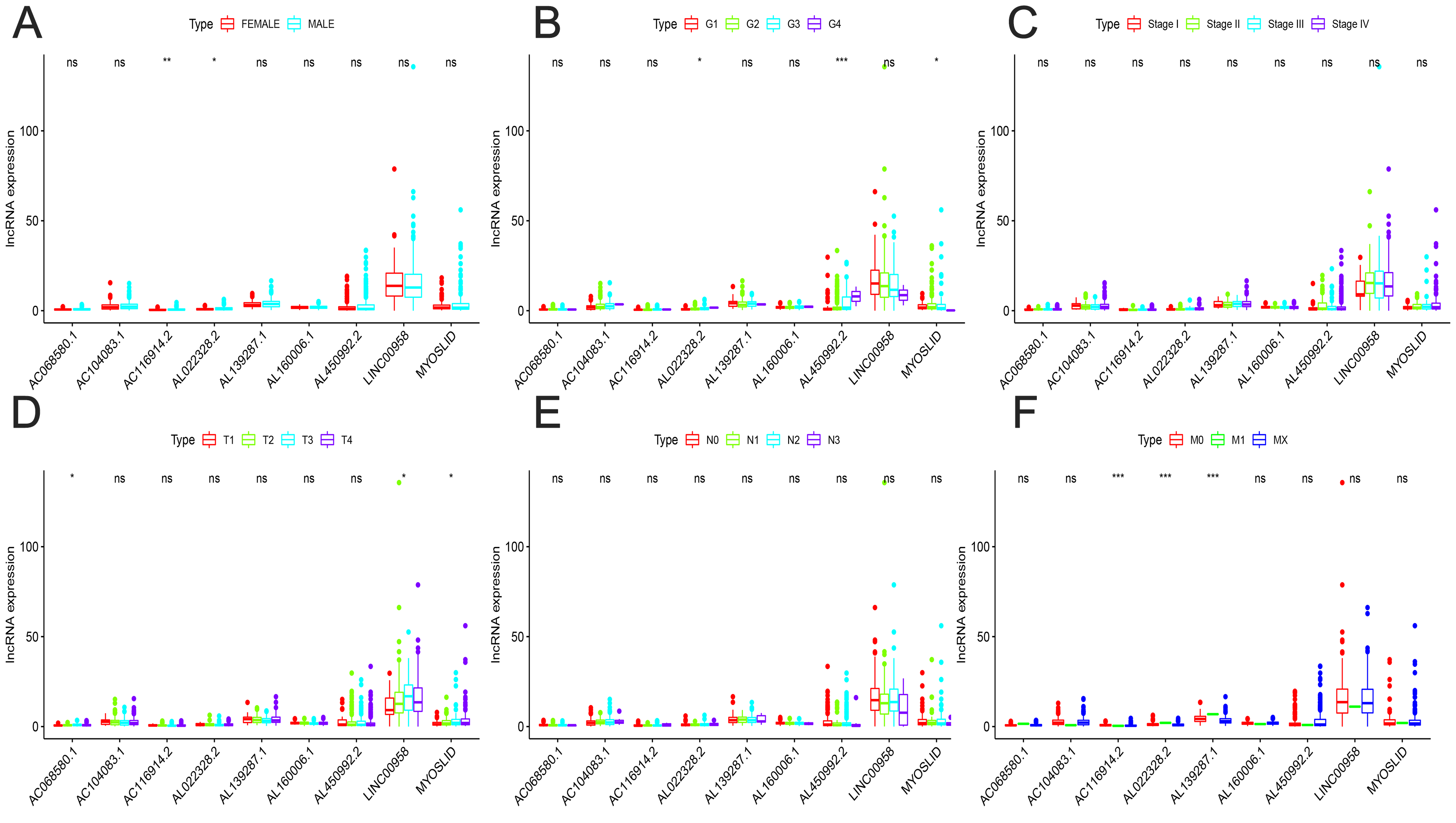

Supplement: Supplementary Figure 2 — Relation between autophagic signature lncRNAs and clinical parameters. (A) gender (female vs. male); (B) grade (G1, G2, G3, G4); (C) AJCC Stage (Stage I, II, III, IV); (D) T stage (T1, T2, T3, T4); (E) Stage N (N0, N1, N2, N3); (F) Stage M [M0, M1, MX (unknown)]. [file Image_2.tif]

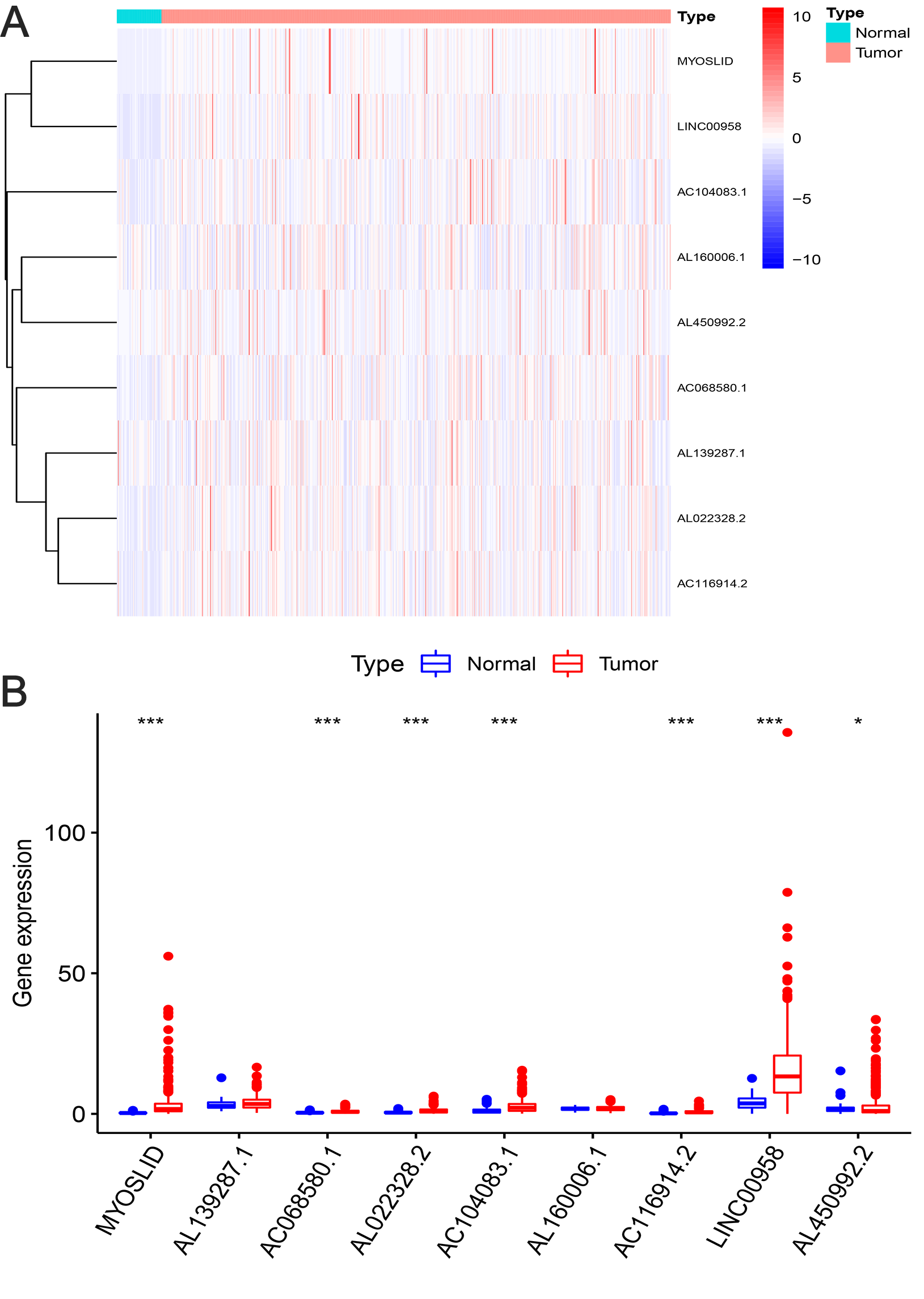

Supplement: Supplementary Figure 3 — Expression levels of signature lncRNAs between normal tissues (n=44) and tumor tissues (n=502). (A) heatmap and (B) boxplot. [file Image_3.tif]
